# Strontium hexaferrite platelets: a comprehensive soft X-ray absorption and Mössbauer spectroscopy study

**DOI:** 10.1038/s41598-019-48010-w

**Published:** 2019-08-13

**Authors:** G. D. Soria, P. Jenus, J. F. Marco, A. Mandziak, M. Sanchez-Arenillas, F. Moutinho, J. E. Prieto, P. Prieto, J. Cerdá, C. Tejera-Centeno, S. Gallego, M. Foerster, L. Aballe, M. Valvidares, H. B. Vasili, E. Pereiro, A. Quesada, J. de la Figuera

**Affiliations:** 1 Instituto de Quimica Física “Rocasolano”, CSIC, Madrid, E-28006 Spain; 2 Institut “Jozef Stefan”, Ljubljana, 1000 Slovenia; 3 Universidad Autónoma de Madrid, Madrid, E-28049 Spain; 4 Instituto de Ciencias de Materiales de Madrid, CSIC, Madrid, E-28049 Spain; 5 Alba Synchrotron Light Facility, CELLS, Barcelona, E-08290 Spain; 6 Instituto de Cerámica y Vidrio, CSIC, Madrid, E-28049 Spain

**Keywords:** Magnetic properties and materials, Surfaces, interfaces and thin films

## Abstract

Platelets of strontium hexaferrite (SrFe_12_O_19_, SFO), up to several micrometers in width, and tens of nanometers thick have been synthesized by a hydrothermal method. They have been studied by a combination of structural and magnetic techniques, with emphasis on Mössbauer spectroscopy and X-ray absorption based-measurements including spectroscopy and microscopy on the iron-L edges and the oxygen-K edge, allowing us to establish the differences and similarities between our synthesized nanostructures and commercial powders. The Mössbauer spectra reveal a greater contribution of iron tetrahedral sites in platelets in comparison to pure bulk material. For reference, high-resolution absorption and dichroic spectra have also been measured both from the platelets and from pure bulk material. The O-K edge has been reproduced by density functional theory calculations. Out-of-plane domains were observed with 180° domain walls less than 20 nm width, in good agreement with micromagnetic simulations.

## Introduction

Strontium ferrite (SFO, SrFe_12_O_19_), isostructural to magnetoplumbite (space group P6_3_/mmc) has a large magnetocrystalline anisotropy. Since its discovery in the mid-20th century, this hexagonal ferrite has become an increasingly important material both commercially and technologically, finding a variety of uses and applications because of its low cost and toxicity. SFO has been used for permanent magnets, recording media, in telecommunications, and as a component in microwave, high-frequency and magneto-optical devices^1,2^. It belongs to the M-type ferrites^3^, together with barium ferrite, and it was first manufactured in the 1960s by Philip’s laboratories^4^.

**Figure 1:**
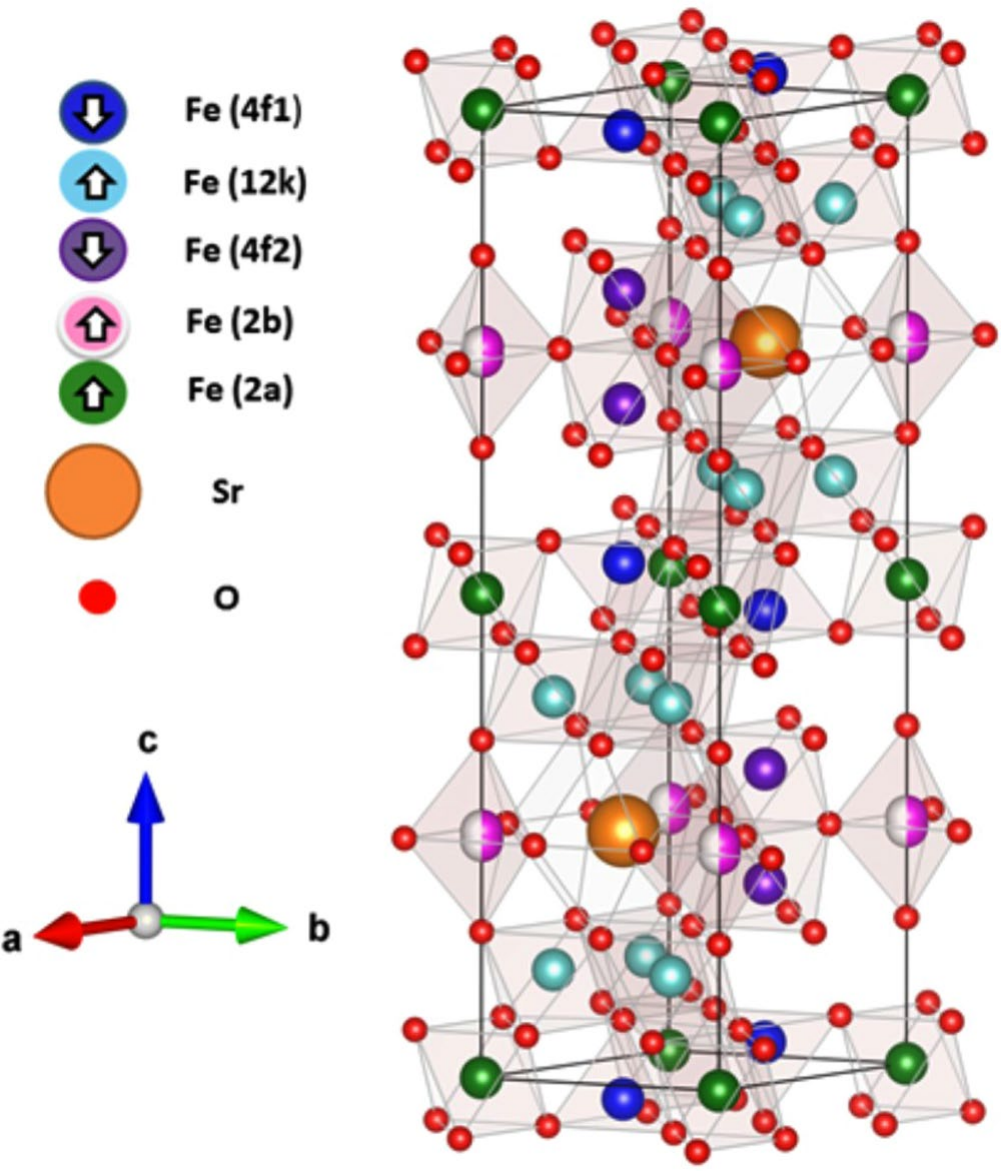
Crystallographic structure of (SrFe_12_O_19_)_2_. This figure was made through TESTA program^63^.

SFO is ferrimagnetic with a Curie temperature of 732 K and a typical magnetic moment in the range of 17–21 *μ*_*B*_ per formula unit^3^. Its structure, shown in Fig. 1, can be considered a sequence of alternating spinel (S) and rocksalt (R) blocks. All the iron cations are in the Fe^3+^ oxidation state, with five different cation environments which correspond to the 2a, 2b, 4f1, 4f2, and 12k positions in the Wickhoff notation^3^. At the spinel block, four octahedral iron cations (2a and 12k) point towards the net magnetization direction, while two tetrahedral (4f1) ones are antiferromagnetically oriented. In the R block, the presence of the Sr^2+^ distorts the neighboring octahedral iron sites, giving rise to two distorted octahedral sites (4f2) which are antiferromagnetically coupled to the rest of the octahedral sites (12k). It also has an unusual bipyramidal Fe site (2b), coupled ferromagnetically to the majority of octahedral sites. As in other M-ferrites the easy axis lies along the c-axis^5–7^. It presents a high magnetocrystalline anisotropy of K_*U*_ = 3.6 · 10^5^ Jm^−3^ ^8^. However, some of its magnetic properties can be affected to a large extent by the shape and size of the material particles and, in the particular case of thin films, by their thickness^9–11^.

Several methods have been used to prepare strontium ferrite such as solid-state synthesis, physical vapour deposition, ball milling, sol-gel and chemical coprecipitation^7,12–17^. In particular, hydrothermal methods can provide highly crystalline micrometer sized platelets^18–20^.

In this work, we have studied the properties of single-crystal platelets of SrFe_12_O_19_ synthesized by the hydrothermal method. We have characterized the structural and magnetic properties of these platelets by Mössbauer spectroscopy, x-ray transmission microscopy (TXM), transmission electron microscopy (TEM), x-ray diffraction (XRD), vibrating-sample magnetometry (VSM), x-ray absorption spectroscopy (XAS), x-ray circular magnetic dichroism (XMCD) and photoemission electron microscopy (PEEM). To the best of our knowledge this is the first time that the x-ray absorption spectra at the Fe L_2,3_ edges of this material in its pure form have been reported. The experimental results have been complemented with multiplet calculations^21^ aimed at reproducing the observed XAS and XMCD spectra at the Fe L_2,3_ absorption edge^22,23^, and by density functional theory (DFT) calculations to reproduce the oxygen K-absorption edge as well as to estimate the iron magnetics moments. Finally the domain pattern measured in remanence is compared with micromagnetic simulations.

## Results and Discussion

**Figure 2:**
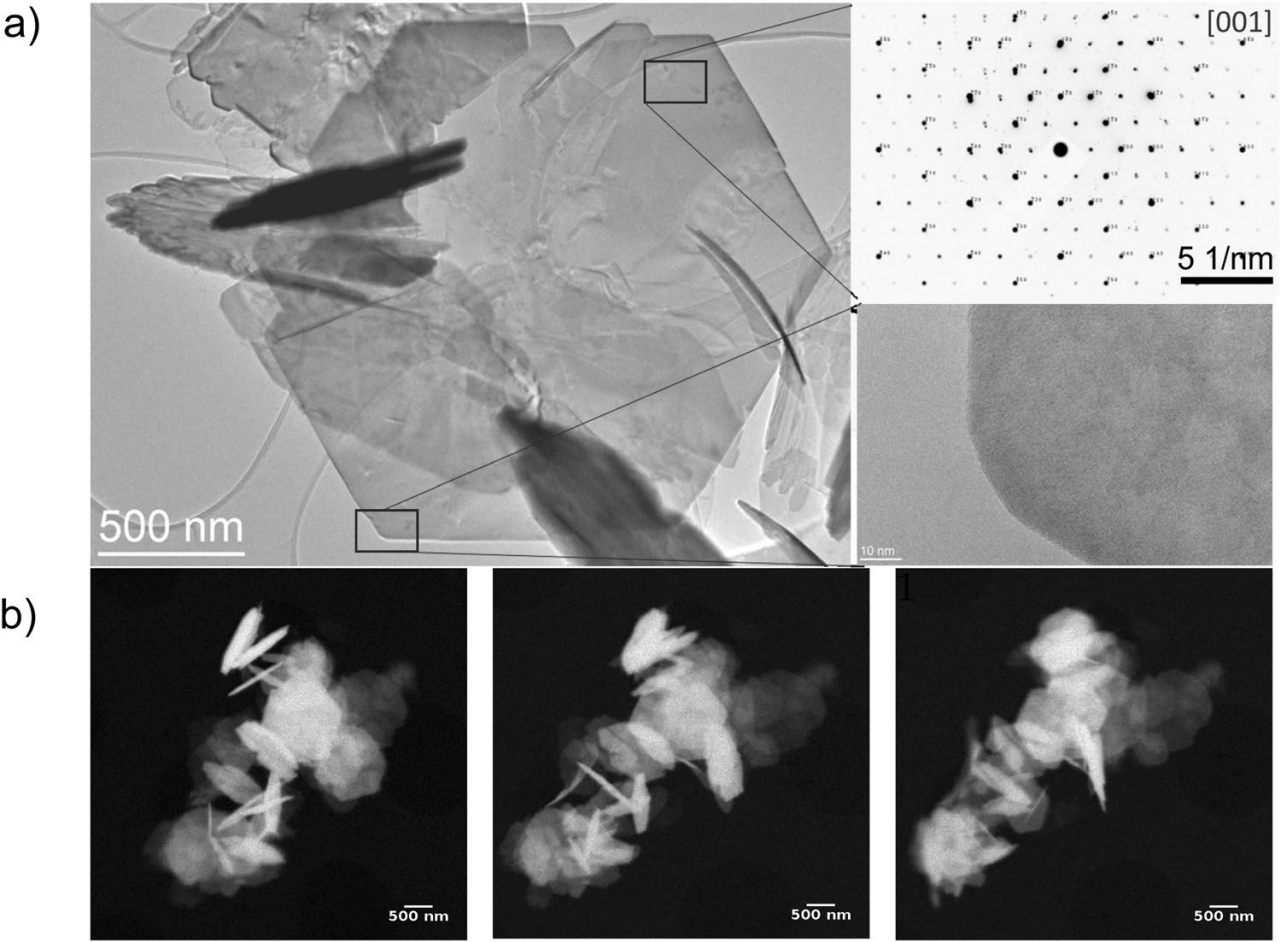
(**a**) TEM images and SAED of SFO particles synthesized at 503 K. (**b**) TXM images taken with a Fresnel zone plate of 25 nm showing the absorption contrast of the same cluster of SFO particles, at three different angles (−30°, 0°, 30°). Effective pixel size = 10 nm.

The hydrothermal method can provide under appropriate conditions highly perfect materials. In order to check the morphology and crystallinity of the as-synthesized powders, they were examined under electron (Fig. 2a) and x-ray absorption microscopy (Fig. 2b) techniques. The TEM examination of the as-synthesized SFO powders revealed a few small particles, below 100 nm, and a majority of platelets with lateral dimensions around 1 *μ*m and thickness of a few tens of nanometers. The platelets present a typical hexagonal shape and observable lattice fringes at higher magnifications (Fig. 2a), which suggests a reasonable crystallinity. This deduction was confirmed by selected area electron diffraction (SAED). For the identification of the SAED pattern, the reference diffraction pattern for SrFe_12_O_19_ simulated with SingleCrystalTM using 69022 ICSD file was used. The SAED result is compatible with the SrFe_12_O_19_ crystal planes being oriented in the [001] direction (Fig. 2a). TXM images were acquired in absorption contrast at the Fe L_3_ and L_2_ edges, Fig. 2b. Each panel corresponds to the same area rotated by 30° with respect to the Y axis. With these images we can appreciate that the platelets are oriented randomly in the three directions and that they form clusters due to their interparticle magnetic interactions.

**Figure 3:**
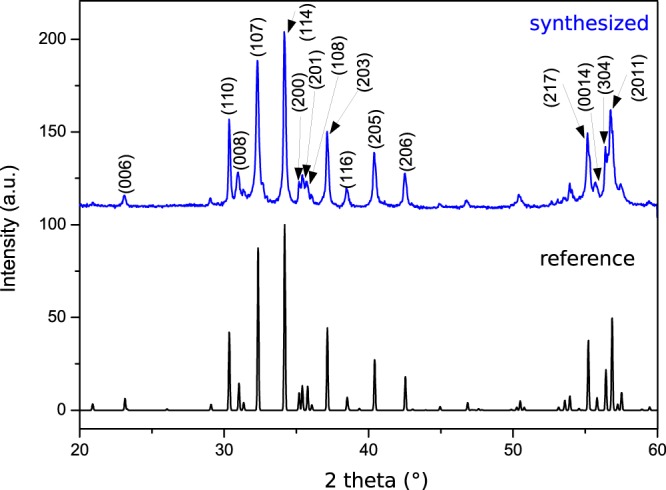
XRD diffraction patterns from the SFO particles synthesized at 503 K (top spectrum), together with the reference pattern (bottom one).

The SFO platelets were characterized structurally by XRD and Mössbauer spectroscopy. Figure 3 shows the XRD diffraction pattern of the SFO platelets and the reference diffraction pattern for SrFe_12_O_19_ simulated with Crystal DiffractTM using the 69022 ICSD file. All the diffraction peaks of the as-synthesized SFO platelets can be indexed according to the reference hexagonal structure of SrFe_12_O_19_. No additional peaks were detected indicating the absence of secondary phases. However, the broadening of the peaks anticipates that the XRD data can be affected by size/thickness effects.

**Figure 4:**
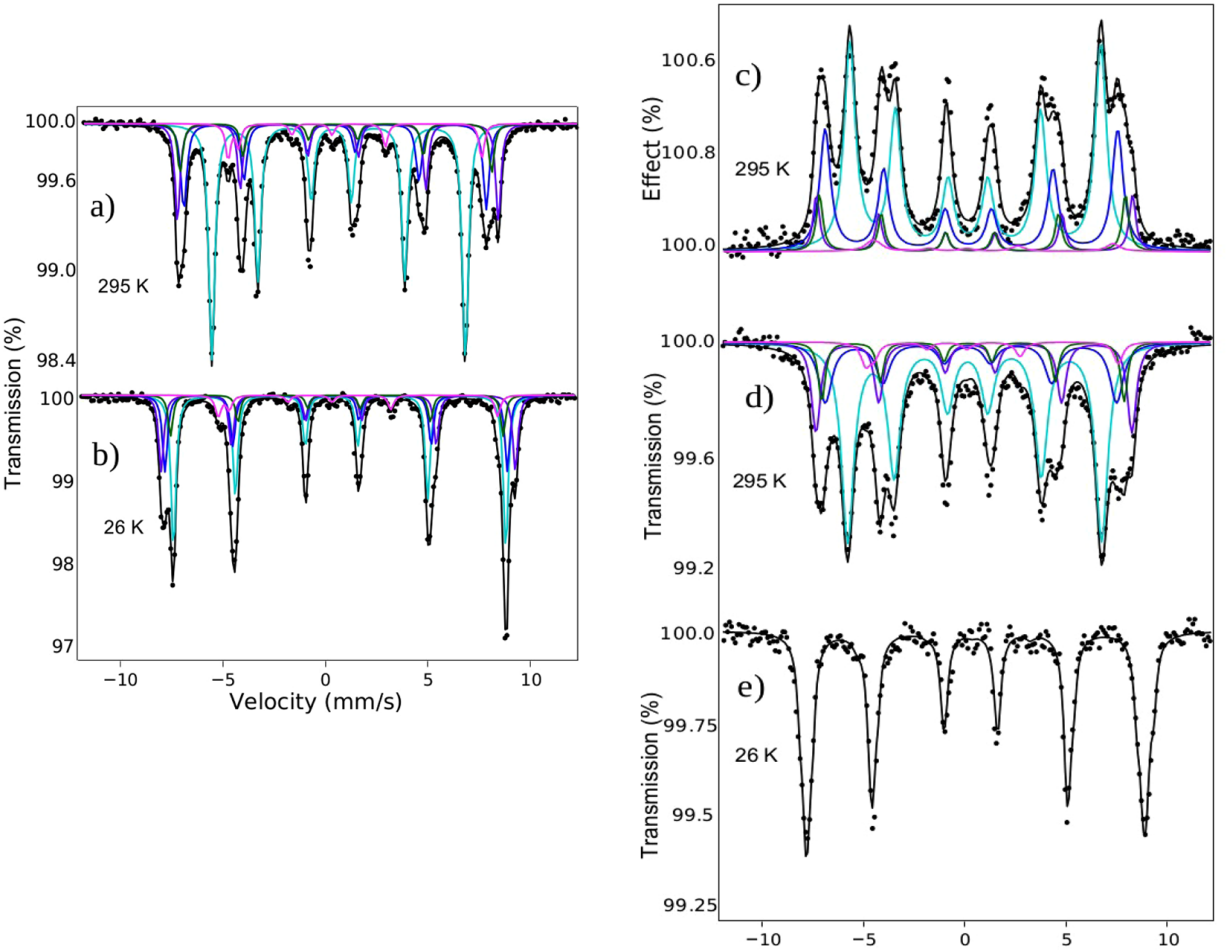
(**a-b**) Mössbauer spectrum of SFO commercial in transmission at 295 K and at 26 K, respectively. (**c**–**e**) Mössbauer spectrum of the SFO platelets in electron detection mode at 295 K and transmission mode at 295 K and at 26 K, respectively. The five sextets in each spectrum correspond to the sites where the iron cations are positioned.

Mössbauer spectroscopy provides information about the oxidation state and coordination of iron. The room temperature Mössbauer spectrum of strontium hexaferrite (SFO) is quite complex as it contains five overlapping sextets each one associated to a specific Fe^3+^ crystallographic site in the SFO structure. Because of this complexity, there is some dispersion in the hyperfine parameters which characterize these sextets^24^. So, in this work we have recorded the room temperature (RT) and 26 K spectra from a pure commercial strontium hexaferrite sample in order to have an inner standard which can guide us in the fit of the Mössbauer spectra recorded from the SFO platelets which are the specific matter of this investigation. These two spectra are shown in Fig. 4a,b, and the corresponding hyperfine parameters obtained from the fit to a sum of sextets having Lorentzian shape lines of the RT data are collected in Table 1. The obtained hyperfine parameters and spectral areas are all reasonably within the range of those reported previously for this material^25–27^. The 26 K spectrum has a very different shape resulting from the increase in both the hyperfine magnetic field values (whose temperature variation is not the same for each of the different sites)^27^ and the isomer shifts of the various contributions. Similarly to that described in ref.^27^, this spectrum has been fitted to five sextets (Fig. 4b), the results being also in reasonable agreement with these literature values.

**Table 1:** ^57^Fe Mössbauer parameters obtained from the fit of the spectra recorded in Fig. 4 at 295 K, being SFO commercial (C), SFO platelets (P) and SFO platelets measured by ILEEMS (P - ILEEMS).

Sample	Site	*δ* (±0.03 mms^−1^)	2 *ε* (±0.05 mms^−1^)	H (±0.01T)	Γ (±0.03 mms^−1^)	Area %
C - 295 K	12k	0.35	0.38	40.5	0.38	49
	4f1	0.26	0.18	48.3	0.38	18
	4f2	0.38	0.26	51.3	0.32	18
	2a	0.33	0.14	49.8	0.32	9
	2b	0.27	2.22	40.5	0.32	6
P - 295 K	12k	0.35	0.38	40.6	0.78	53
	4f1	0.25	0.22	46.9	0.66	17
	4f2	0.39	0.26	50.3	0.68	17
	2a	0.31	0.14	49.3	0.52	9
	2b	0.26	2.32	40.2	0.56	4
P - ILEEMS	12k	0.35	0.38	40.8	0.72	53
	4f1	0.26	0.16	47.5	0.70	29
	4f2	0.40	0.22	51.4	0.42	8
	2a	0.33	0.14	49.7	0.42	8
	2b	0.26	2.32	39.3	0.80	2

The symbols *δ*, 2 *ε*, H, Γ correspond to isomer shift, quadrupole shift, hyperfine magnetic field and linewidth, respectively.

The RT Mössbauer spectrum recorded from the SFO platelets is depicted in Fig. 4d. Compared to that of the standard sample, this spectrum shows much broader lines (about twice broader than in the SFO reference) and resembles other spectra recorded from SFO samples made by hydrothermal synthesis^26,27^. This might be related to the occurrence of disorder or structural inhomogeneities in the material^25^. The broadening of some of the lines in the x-ray diffraction data (see Fig. 3) is compatible with this interpretation. An explanation of the line broadening considering the occurrence of superparamagnetic effects could be discarded as the lateral dimensions of the platelets are in the micrometer range. The spectrum fits well considering a 3:2:1:1:2:3 area ratio for the lines of all the sextets indicating that the platelets in the sample are randomly oriented. In any case, the hyperfine parameters obtained from the fit are very similar to those of the SFO standard sample (Table 1).

It is interesting to compare this spectrum with the RT ILEEMS spectrum recorded from the platelets (Fig. 4c). In an ILEEMS spectrum the surface contributions are enhanced^28^, therefore the differences, if any, between the ILEEMS and transmission spectra have to be due to structural/compositional changes in the surface respect to the bulk. Inspection of Fig. 4c,d show clear differences between these two spectra, particularly in the outermost lines. The fit to the ILEEMS spectra shows a considerable increase of the intensity of the area of the sextet corresponding to the tetrahedral 4f1 sites which almost doubles respect to that shown in the transmission spectrum (Table 1). Therefore, the results point out to a higher concentration of tetrahedral sites at the surface. At this respect is also very interesting to compare the 26 K spectrum recorded from the platelets (Fig. 4e) with that recorded at that temperature from the SFO standard. Again, there is a large difference between these two spectra as it can be appreciated in Fig. 4: the spectrum of the platelets is less asymmetric and the outer lines are broader. We must recall that there is no unique fit to this low temperature platelets spectrum. It is clear that the lines are much narrower than in the RT spectrum and, therefore, that the strong overlap between the different contributions complicates the fit. In the case of the spectrum of the SFO standard this difficulty is mitigated at some extent because the outer sextets are relatively well distinguished from that corresponding to the most populated 12k site (see outer lines of the spectrum in Fig. 4b). Consequently, we have decided not to give any particular fitting model for the 26 K spectrum of the platelets but to make qualitative comments only. When trying to fit this spectrum we have observed various trends: (i) there is a strong tendency to obtain as the most intense sextet the one having hyperfine parameters close to tetrahedral coordination; depending on the parameters fixed, the intensity of the sextet corresponding to site 2a increases noticeably but its isomer shift goes to very low values again compatible with a coordination lower than the octahedral one which is the one expected (it is known that the hyperfine parameters of sites 4f1 and 2a are strongly correlated)^24^ and (ii) the area of the sextet corresponding to the octahedral site 12k has a tendency to decrease; in some of the fitting models tried the area goes down to a half of the expected value. The large increase of the contribution corresponding to “tetrahedral/lower than octahedral coordination sites” at the 26 K spectrum of the platelets might be understood on the basis of their recoil free fraction. Since, as the ILEEMS data have suggested, these sites are preferentially located at the surface of the platelets it could be assumed that their recoil free fraction at room temperature is low^29^ and that it increases dramatically at low temperature.

Taken together the results seem to indicate that the platelets contain a large amount of “tetrahedral/lower than octahedral coordination sites” which are mainly located at the surface. It must be taken into account that given the shape of the platelets, whose lateral dimensions are several orders of magnitude larger than their thickness, the amount of sites unsaturated in oxygen which are located at the surface has to be overwhelming as compared to the number of these sites in the bulk. It would follow that the broadening observed in the XRD data, and at some extent in the RT Mössbauer data, would reflect then the various configurations arising from the distribution of iron ions which cannot complete their octahedral coordination and that can show either tetrahedral- or penta-oxygen coordination. As the low temperature data have suggested, this situation would imply, consequently, a reduction in the number of well-defined 12k sites existing in the platelets. This should be reflected in the magnetic moment measured in these particles (see below).

**Figure 5:**
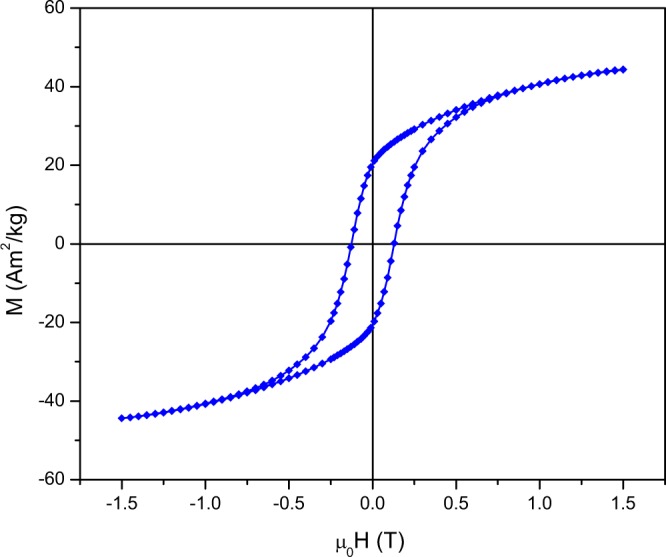
Room-temperature hysteresis loops of as-synthesized SFO particles.

The room-temperature hysteresis cycle of the platelets is presented in Fig. 5. The maximum and remanent magnetization values are 44.4 Am^2^/kg and 20.4 Am^2^/kg, respectively. The remanent-to-saturation magnetization ratio is 0.46, which can imply some slight preferential orientation of the particles, induced by the sample preparation. That the magnetization at 1.5T is lower than the usual bulk values of saturation magnetization can be attributed to a combination of nanosize effects and an applied field that does not fully saturates the platelets. Namely, it is known that in nanoparticles the high surface-to-volume ratio results in the formation of a magnetically dead surface layer that causes spin canting which leads to a decrease in the saturation magnetization and remanence^30^. The coercivity of as-synthesized platelets is 0.13T, which is also lower than the bulk coercivity of SrFe_12_O_19_. This again maybe a consequence of the 1.5T employed as maximum applied field, as the anisotropy field of SFO is of the order of 1.8T^3^. In addition, being an extrinsic property, coercivity is directly related to the particle size. To increase the coercivity as much as possible, one should be in the very narrow size range close to the critical diameter for single-domain particles, which for SrFe_12_O_19_ ranges from 0.6 *μ*m to 1 *μ*m. This range can be tuned by tailoring the Fe/Sr ratio in the starting precursors^10,31^.

**Figure 6:**
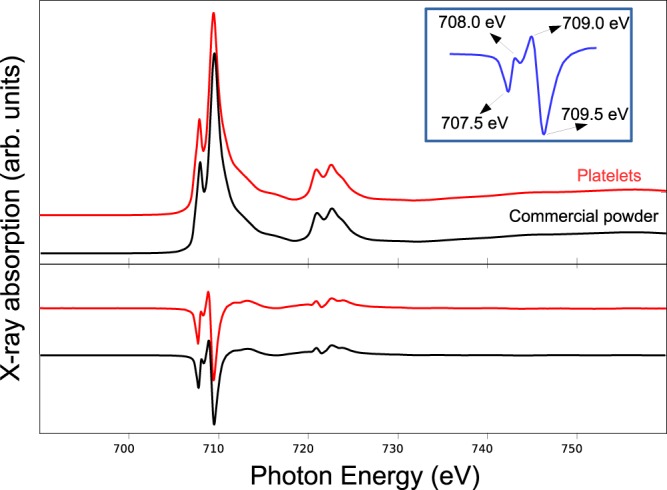
X-ray absorption spectra at the Fe L_2,3_ absorption edges recorded from the platelets and the commercial powder, they were acquired at 2 K under an applied field of 6T and averaged for both light helicities. Bottom: x-ray magnetic circular dichroism spectra for both temperatures obtained by subtracting the spectra acquired with opposite light helicities. Inset: Energy positions for each of the peaks observed in the Fe L_2,3_ edge XMCD spectra.

To probe both the structure and the magnetic properties of SrFe_12_O_19_ we resort to x-ray absorption and magnetic circular dichroism. Spectra were acquired under a 6T magnetic field to saturate the magnetization of the sample, both at room temperature and at 2 K and at the iron L_3_ and L_2_ edges and the oxygen K edge. The x-ray iron absorption spectrum, presented in Fig. 6, shows at both L_3_ and L_2_ edges the typical double peak structure characteristic of Fe^3+^. Further information can be gleaned from the x-ray magnetic circular dichroism spectrum (see lower panel of Fig. 6). We also recorded for reference the spectrum of the SrFe_12_O_19_ powder from a commercial supplier^32^. The spectrum from the commercial powder and that of our hydrothermal samples are similar. The iron XMCD spectrum presents several characteristic features, of which the most prominent at the L_3_ edge is an initial large valley (707.5 eV), then a small peak (708.0 eV) and valley structure followed by a peak (709.0 eV) and a large valley (709.5 eV). Other spectra acquired from different SFO samples (thin films, other hydrothermal platelets) provided the same features in their spectra (not shown).

The main peak at 709.0 eV arises mostly from Fe^3+^ in tetrahedral positions, while the last, deepest, valley originates mostly from Fe^3+^ in octahedral positions. However, all the SrFe_12_O_19_ spectra we measured also show the first valley (707.5 eV), which in iron oxides can be attributed to Fe^2+^ in octahedral positions^33–35^ or to Fe^3+^ in octahedral sites^36,37^. Taking into account that there is no Fe^2+^ in our material, as shown by Mössbauer spectroscopy, we present now a multiplet calculation of the contributions of the different cation environments for Fe^3+^ ^21^. Calculations were performed with the Crispy + Quanty code^38,39^ using octahedral crystal field, tetrahedral one and for the bipyramidal site, C3v crystal field symmetry.

**Figure 7:**
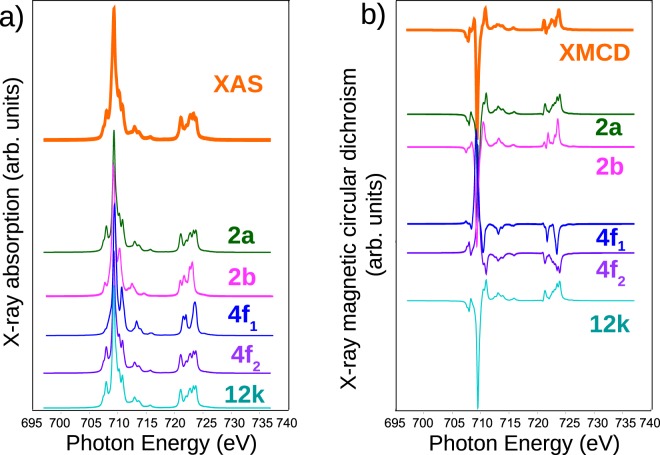
Atomic multiplet calculations used to simulate the XAS and XMCD spectra at the Fe L_2,3_ absorption edges. For each iron cation, the XAS and XMCD spectra are calculated with the proper crystal field and we show on the top of each figure the sum of the sublattice contributions.

The differences in the oxygen-cation distances^40^ for the different octahedral environments are not expected to change significantly the crystal field splitting. Thus, we have used the same 10Dq parameter (=1.1 eV) for all octahedral sites (2a, 4 f_2_, 12k). In addition, we have modeled the tetrahedral sites(4 f_1_) with 10Dq = −0.5 eV, and the bipyramidal one(2b) with 10Dq = 0.8 eV, D *τ* = −0.08 eV and D *σ* = 0.01 eV using literature values^22^. The Slater parameters were reduced to 72% of the Hartree Fock values following ref. ^22^. The Gaussian broadening value used for each peak in the simulated spectrum was 0.09 eV, in order to match the experimental resolution. Each component as well as the complete fit are shown in Fig. 7. The overall fit is reasonable.

In Fig. 7a the shape of the XAS spectrum is seen to be due mainly to the contributions of iron in octahedral positions. However, the origin of features of the XMCD spectrum is more involved. The spectrum of maghemite, which only has Fe^3+^ in an spinel structure, is quite similar to our strontium hexaferrite spectrum but lacks the small peak at 708.0 eV^41^. So one possibility is that this peak arises from the Fe^3+^ in a bipyramidal environment, which is missing in maghemite. However, there is only one atom per formula unit in that environment. Actually, the Mössbauer spectrum already indicated the small contribution of this site. To check the role of the bipyramidal site in the XMCD spectrum, we added the different cation contributions without this position: the peak at 708.0 eV still appeared. In fact, both maghemite and ferrihydrite have also been reported to present that peak in high resolution experiments^36,37^. Therefore, we conclude that the Fe^3+^ cations in octahedral and tetrahedral environments are the ones responsible for the observed features of the XMCD spectra.

**Figure 8:**
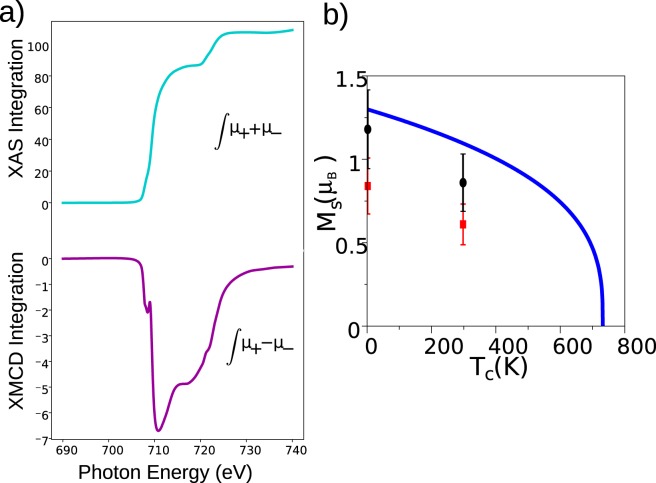
(**a**) Integral of XAS and XMCD used to estimate the orbital and spin magnetic moment of Fe in SFO. (**b**) Total magnetic moment per Fe cation for the platelets (red squares) and from commercial SFO powder (black circles) for two temperatures. We take into account an error in the sum rules of 20% for the calculation of spin orbital moment^64^. The continuous line is the expected change of the magnetization from the bulk magnetic moment with a critical exponent 0.33, which corresponds to a 3D Ising model^46^.

The sum rules^42^ can be used to estimate the spin and orbital contributions to the magnetic moment of the iron cations from the XMCD spectra (see Fig. 8a). In particular, they provide the spin moment together with the expected value of dipolar operator. However that contribution is negligible for Fe^3+^ (d^5^)^43^. In order to estimate not only the ratio of the spin to the orbital moment, but their absolute magnitude, the number of d-holes for the cations is required. We set it to 4.7 following ref.^44^. The application of the sum rules gives 0.84 *μ*_*B*_ and 0.01 *μ*_*B*_ for the spin and orbital moments for the platelets at 2 K, respectively. The spin magnetic moment of the commercial powder at the same temperature is 1.23 *μ*_*B*_ and its orbital magnetic moment is 0.04 *μ*_*B*_.

The difference found between the platelets and the commercial powder can be rationalized with the information provided by the Mössbauer spectra (see Fig. 4c–e). They show (as we discussed previously) that the platelets have more iron cations in either tetrahedral or lower-than-octahedral coordination sites. While the 12k octahedral sites point in the net magnetization direction of the SFO, the 4f1 tetrahedral cations point in the opposite direction. Therefore, the variation of the cation population detected in Mössbauer suggests a lower spin magnetic moment in the platelets.

Our DFT calculations give a total magnetic moment per formula unit of 20.00 *μ*_*B*_, in close agreement with previous calculations^45^. The Bader charges and magnetic moments projected on the different Fe cations are given in Table 2. As usual in DFT-based calculations, we obtain fractional ion states ranging between +1.54 (2b) and +1.71 (4f2) well below the Fe^3+^ configuration generally assumed for this material. For all the iron cations, the calculated magnetic moment is close to 4.0 *μ*_*B*_. If we neglect the magnetic moments (MMs) induced in the oxygen anions, the total magnetic moment per f.u. is 17.2 *μ*_*B*_, giving an average of 1.39 *μ*_*B*_ per Fe cation. This calculated value is in reasonable agreement with the experimental one obtained from the commercial powder.

**Table 2:** Bader charge and magnetic moment of each Fe site and total values per formula unit including also the oxygen and Sr ions.

Site	Charge (e)	MM (*μ*_*B*_)
12k	6.37	4.07
4f1	6.41	−3.96
4f2	6.29	−3.93
2a	6.32	4.08
2b	6.46	3.99
TOTAL	220.00	20.00

The magnetic moment per cation for both the platelets and the commercial powder decreases with temperature (see Fig. 8b). The decrease is compatible with a critical exponent *β* of 0.33 for the magnetization *vs* temperature dependence (T_c_ − T)^*β*^. This critical exponent is expected from the 3D Ising model^46,47^, where the magnetization vector can only lay along two directions in the structure (up and down in Fig. 1). This is reasonable in view of the strongly uniaxial character of the strontium hexaferrite detected through the vector analysis from XMCD-PEEM images discussed below.

**Figure 9:**
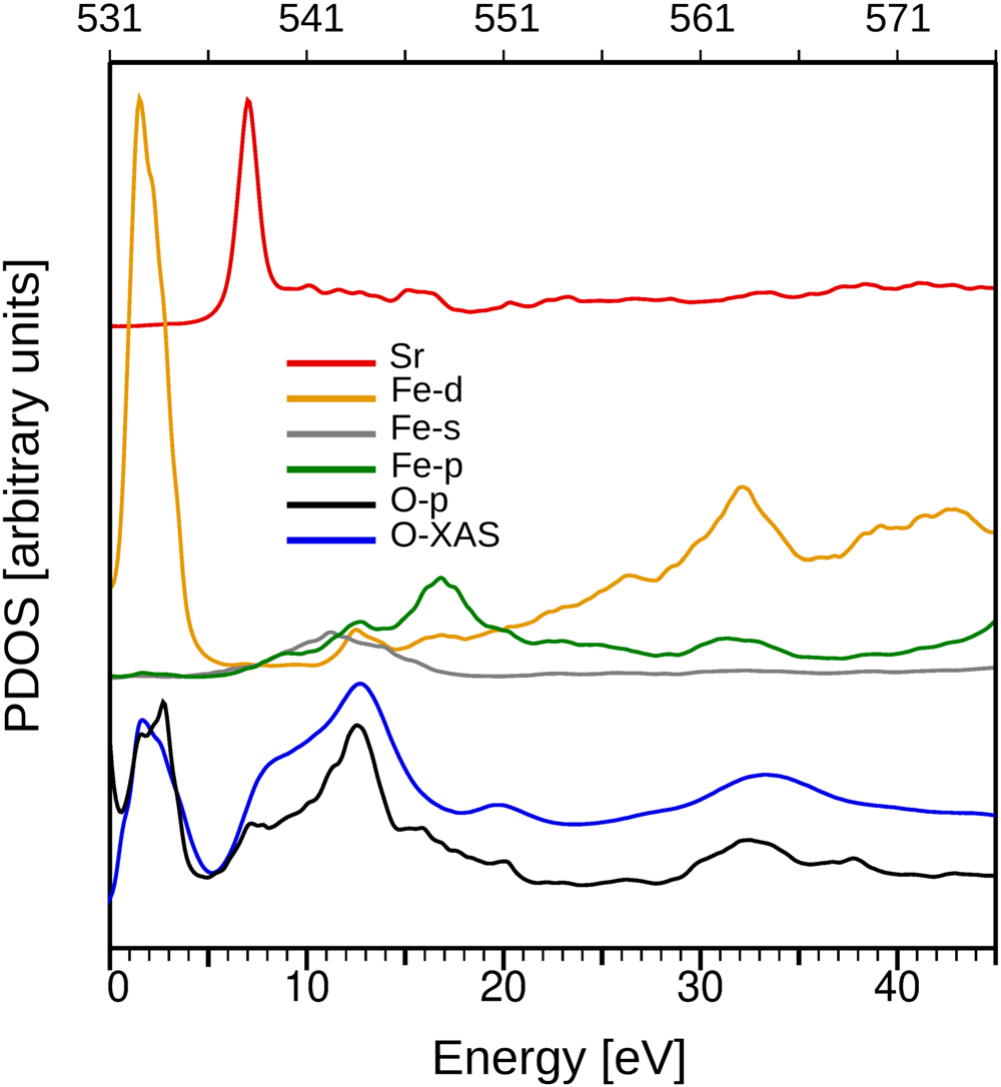
Blue line: X-ray absorption spectrum at the O K absorption edge, recorded at 2 K and an applied magnetic field of 6T from the SFO platelets and averaged for both light helicities. Rest of lines: Calculated density of states projected on the Sr atoms, Fe-*d*, *p* and *s* and the O-*p* states (see legend). The PDOS curves for each species have been vertically shifted and rescaled for visual inspection.

The O K-edge corresponds to transitions from the oxygen 1 s to the oxygen unoccupied states, which are 2p orbitals strongly hybridized with the iron ones. The spectrum, shown in Fig. 9 (blue line) presents several well defined peaks which can be compared against equivalent data measured for different iron oxides^48,49^. In order to interpret the O-K XAS spectra, it is common to disregard the influence of the core hole on the unoccupied bands, as these have most weight on the metal sites^50^. This allows to consider the peaks directly as arising from the unoccupied density of states of the oxygen. Following refs^48,50^, the double peak at 532 eV is explained by the hybridization of 2p antibonding oxygen states with iron 3d states. The separation between the two peaks that compose it is related to the splitting between the t2g and eg orbitals in the iron cations due to the crystal field^50^. However, as is the case for maghemite and magnetite^49^, the presence of three different iron environments (tetrahedral, octahedral, and trigonal bipyramidal) smears out the clear double peak detected in hematite. The peaks at higher energies (536–576 eV) reflect transitions into oxygen p-states hybridized with extended 4p and 4 s iron states. Their particular origin has been assigned by means of multiple scattering cluster calculations in ref.^48^, to intrashell multiple scattering (peak at 545 eV) and single scattering between the absorber and consecutive oxygen shells (peaks at 563 and 550 eV). In Fig. 9 we compare the XAS spectrum with the density of unoccupied states projected (PDOS) on the O 2p state directly calculated by DFT (dark line). Most of these peaks are correctly reproduced both in shape and energy location within the expected accuracy of the DFT formalism. Furthermore, comparison versus the DOS projected on the Sr atoms and the Fe-*s*, -*p* and -*d* states reveals that the influence of the formers on the oxygen p-states is negligible compared with the latter and that, particularly at higher energies, there is certain correlation between the O-*p* and Fe-*p* PDOS.

**Figure 10:**
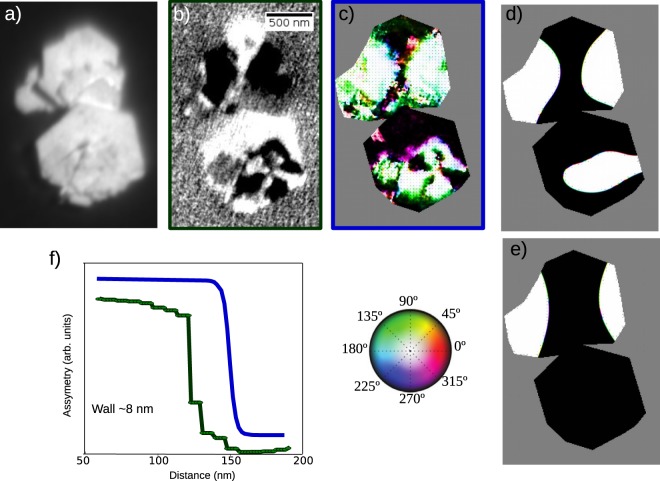
(**a**,**b**) XAS and XMCD images, respectively. (**c**) Magnetization vector in the platelets pixel by pixel. (**d**) Relaxed initial configuration calculated considering literature parameters for this compound, (**e**) Relaxed initial configuration using in the simulation experimental values and (**f**) comparison between domains wall width of the experiment and simulations.

We note that the measurements shown in Fig. 6 comprise a large quantity of particles. In order to perform absorption experiments on a single platelet, we made use of XAS and XMCD in a PEEM microscope. This allows determining the remanence vector magnetization pattern, as shown in Fig. 10. First, pairs of XAS images have been acquired with opposite x-ray helicities, by measuring the spatially resolved emission of secondary electrons at low kinetic energy. Then both images are added and subtracted pixel-by-pixel to provide the averaged XAS image (Fig. 10a) and XMCD image (Fig. 10b), respectively. When a full spectrum is required, a stack of images acquired at different photon energies is measured, and the intensity on the screen in the desired area is integrated for each image.

The averaged XAS image (Fig. 10a) shows platelets that appear partially stacked on top of each other. There is a detectable shadow in some of them, allowing estimating their thickness as 18 nm. The asymmetry contrast is proportional to the local magnetization along the x-ray beam direction^51^, which in this case is partially out-of-plane, with a fixed polar angle of 74° (where 90° indicates the in-plane direction). In order to determine the magnetization vector, images with three non-coplanar orientations^52^ of the x-ray beam relative to the sample were measured (not shown). From their combination, the pixel-by-pixel magnetization vector can be determined, as shown with a color map in Fig. 10c. The platelets in the image are multidomain, with their magnetization vector mostly in and out of the plane (black and white areas in Fig. 10c), with 180° domain walls between them. The domain walls are quite sharp. In fact, their width is likely smaller than the experimental resolution. Their expected width is $\pi \sqrt{A/K}\mathrm{=13}$ nm, where *A* is the exchange stiffness and *K* the uniaxial anisotropy constant^53^. A cut across a domain wall is shown in Fig. 10f, confirming this prediction. The pixel width in the images is 8.5 nm and the overall experimental lateral resolution is around 20 nm^54^.

**Figure 11:**
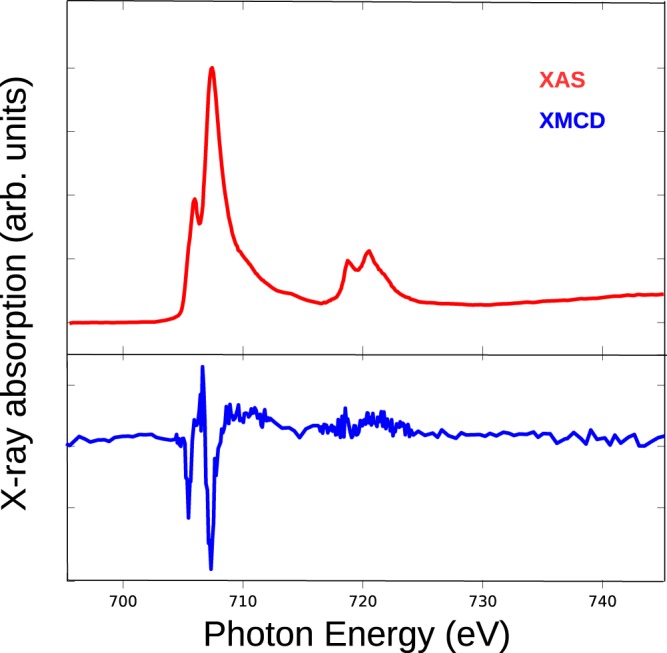
Fe XAS and XMCD spectra acquired from the black domain in the platelets shown in Fig. 10b by PEEM.

Following ref.^52^ we have used the experimental magnetization map as the initial configuration of a micromagnetic simulation for an object with the shape similar to that experimentally determined. The voxel size in the simulation was 4.23 nm, in order to reproduce accurately the domain walls. Using the nominal saturation magnetization for SFO (based on the XMCD measurement of the magnetization moment), and minimizing the initial configuration energy gives rise to the magnetization pattern shown in Fig. 10d. The domains closely resemble the experimental ones, with a similar curvature of the domain walls. A cut across the domain walls gives the magnetization profile shown with a blue line in Fig. 10f. Another simulation was performed using instead the maximum magnetization measured for the platelets by VSM (see Fig. 5e). In this case, the domain walls have less curvature and reproduce worse the experimental results. We believe this is because the VSM magnetization measured at 1.5T underestimates the saturation magnetization. Finally, the absorption and dichroism spectra obtained by XAS and XMCD-PEEM from a single domain are shown in Fig. 11. Although the XMCD spectrum is somewhat noisier than that of Fig. 6, since it is obtained from a sub-micron area at room temperature, they have a one to one correspondence.

## Conclusions

We have synthesized and characterized strontium hexaferrite platelets made by hydrothermal methods using different spectroscopy and microscopy techniques. The platelets are micrometric in lateral size and nanometric in thickness. We present high-resolution XAS and XMCD spectra of the *L*_2,3_ iron absorption edges and oxygen K edge both for the platelets and for the reference commercial material. The iron XAS and XMCD spectra have been reproduced through multiplet calculations, taking into account the different cation environments, while the oxygen spectrum has been compared with density functional calculations. We note that both the XAS and XMCD spectra are remarkably similar to spectra from other Fe^3+^ containing oxides and oxyhydroxides as for example maghemite and ferrihydrite, when acquired with similar resolution. Only Mössbauer spectroscopy has been found to be specific enough to explore the cation distribution, detecting an increase of the tetrahedral sites with respect to the octahedral ones when compared to the reference commercial sample reflected in the reduced magnetic moments determined from the XMCD spectra. By XMCD-PEEM we have imaged the out-of-plane magnetic domains with nanometric resolution and compared them with micromagnetics simulations.

## Methods

SrFe_12_O_19_ particles were synthesized by a hydrothermal method. Aqueous solutions containing the appropriate metal ions were prepared from strontium (II) nitrate (Sr(NO_3_)_2_, 99%+, Across Organics) and iron (III) nitrate nonahydrate (Fe(NO_3_)_3_ × 9 H_2_O, 98%, Carlo Erba Reagents) salts. The exact metal concentration of the reagents was determined by chemical analysis (ICP-AES, PE OPTIMA 3100RL). To the aqueous solution of Sr^2+^ and Fe^3+^, with a Sr^2+^/Fe^3+^ ratio of 1/6, a sodium hydroxide (NaOH, Alfa Aesar, 98%) aqueous solution was added at room temperature so that the final ${{\rm{NO}}}_{3}^{-}$/OH^−^ ratio was $\frac{1}{2}$. The mixture was then put into a stainless-steel autoclave and kept in an oven until a temperature of 503 K was reached for a holding time of 15 min, and immediately after that the heating was turned off and the autoclave was cooled to room temperature.

The SFO platelets were characterized using several microscopy techniques. Transmission electron microscopy (TEM) was performed with a Jeol 2100 instrument using an accelerating voltage of 200 kV. Several types of x-ray microscopy were performed at the iron L_2,3_ edges. In particular, full-field transmission X-ray microscopy (TXM) was carried out at the MISTRAL beamline^55^ and photoemission electron microscopy (XMCD-PEEM) at the CIRCE beamline^54^, both at the Alba synchrotron. In the latter case, an Elmitec SPELEEM instrument was used to image the secondary electrons at a kinetic energy of 2 eV. For TEM measurements, a small amount of as-synthesized powder was dispersed in water and drop-deposited onto a Cu TEM grid. For x-ray microscopies, the powder was dispersed in ethanol, diluted several times, sonicated, and then deposited on a Cu TEM grid (for TXM), or on a Si(100) wafer covered with its native oxide (for XMCD-PEEM).

The crystal structure of the powders was identified by x-ray powder diffraction (XRD) with a Siemens D5000 (Munich, Germany) diffractometer using Cu-K *α* radiation and the EVA software (Bruker AXS, Karlsruhe, Germany). The measuring step was 0.02°/s with a 4 s measuring time per step.

The magnetic properties were studied by acquiring room-temperature hysteresis loops with a vibrating-sample magnetometer using a magnetic field of 1.5 T(VSM; MicroSense EZ7) and by x-ray magnetic circular dichroism spectra, which were measured in total yield mode at a temperature of 2 K and under an applied field of 6T at the BOREAS beamline^56^ of the Alba synchrotron. For the VSM measurement, the powder was placed in a cylindrically shaped powder cup holder, which was tightly sealed with the upper part of the holder, while for the x-ray magnetic circular dichroism spectra the powder was attached to a conducting carbon tape. The experimental energy resolution of the beamline at the measurement conditions was 90 meV.

Mössbauer spectra were recorded in the integral low-energy electron mode (ILEEMS) at 298 K for the platelets and the transmission mode, both for the commercial sample and for the platelets, at 298 K and 26 K using a constant acceleration spectrometer, a ^57^Co(Rh) source, a helium closed cycle cryorefrigerator and a triangular drive waveform^57^. The spectra were computer-fitted and the isomer shift data are quoted relative to the centroid of the spectrum of metallic iron at room temperature. In the case of ILEEMS, we used the same conditions as those described in ref.^58^.

DFT + U based calculations for the (SrFe_12_O_19_)_2_ unit cell were performed employing the VASP^59^ package under the Generalized Gradient Approximation (GGA) to the exchange-correlation functional^60^ including an effective Hubbard term of *U* = 3 eV. The unit cell lattice parameters were set to the experimental values *a* = 5.884 Å and *c* = 23.05 Å (*c*/*a* = 3.917). A cut-off energy of 400 eV was employed for the plane wave basis generation while a (7 × 7 × 5) *k*-supercell was used for the Brillouin sampling. Due to computational limitations, spin-orbit interactions were not included, although their influence on the computed Bader charges and magnetic moments is known to be only marginal. Furthermore, in order to simulate the oxygen unoccupied density of states at energies well above the Fermi level (up to 40 eV) we included up to 1,000 bands in the calculation (parameter NBANDS).

Micromagnetic simulations were performed with the MuMax3 software^61^ using a low-end graphic GPU (GeForce GTX760). The simulations were performed in a slab with a voxel size of 4.23 nm × 4.23 nm × 18 nm to match the pixel size of the experimental images. The hexagonal platelet geometry was taken from the experimental images. The material constants employed for the saturation magnetization, exchange stiffness and magnetocrystalline hexagonal anisotropy were M_*s*_ = 3.8 · 10^5^ Am^−1^, A_*s*_ = 6 · 10^−12^ Jm^−1^ and K_*U*_ = 3.6 · 10^5^ Jm^−3^, respectively^3,7,62^.
